# Multifunctional Nanomaterials for Ferroptotic Cancer Therapy

**DOI:** 10.3389/fchem.2022.868630

**Published:** 2022-03-24

**Authors:** Zhiyuan Shi, Jianzhong Zheng, Wenbin Tang, Yang Bai, Lei Zhang, Zuodong Xuan, Huimin Sun, Chen Shao

**Affiliations:** ^1^ Department of Urology, Xiang’an Hospital of Xiamen University, School of Medicine, Xiamen University, Xiamen, China; ^2^ School of Public Health, Xiamen Univerisity, Xiamen, China; ^3^ Central Laboratory, Xiang’an Hospital of Xiamen University, School of Medicine, Xiamen University, Xiamen, China

**Keywords:** cancer therapy, ferroptosis, nanomaterial, ferroptosis inducers, reactive oxygen species

## Abstract

Patient outcomes from the current clinical cancer therapy remain still far from satisfactory. However, in recent years, several biomedical discoveries and nanotechnological innovations have been made, so there is an impetus to combine these with conventional treatments to improve patient experience and disease prognosis. Ferroptosis, a term first coined in 2012, is an iron-dependent regulated cell death (RCD) based on the production of reactive oxygen species (ROS) and the consequent oxidization of polyunsaturated fatty acids (PUFAs). Many nanomaterials that can induce ferroptosis have been explored for applications in cancer therapy. In this review, we summarize the recent developments in ferroptosis-based nanomaterials for cancer therapy and discuss the future of ferroptosis, nanomedicine, and cancer therapy.

## 1 Introduction

Cancer is one of the leading causes of death and one of the most important diseases affecting human health worldwide (Bray et al., 2021). According to the recent global cancer statistics, there were over 19 million new cancer cases and 10.0 million cancer deaths in 2020, and the cancer burden might be expected to reach 28.4 million cases by 2040 (Sung et al., 2021). Therefore, the development of cancer prevention and treatment strategies is crucial to achieve global cancer control. Conventional approaches, such as surgery, radiotherapy, and chemotherapy, have been utilized; however, the prognosis does not achieve both the doctor’s and patient’s satisfaction due to the hallmarks of cancer, such as sustaining proliferative signaling, evading growth suppressors, nonmutational epigenetic reprograming, avoiding immune destruction, activating invasion and metastasis, inducing vasculature, genome instability and mutation, resisting cell death, and deregulating cellular metabolism (Hanahan 2022). Accompanied with the significant scientific and technological advancements in recent times, several new technologies are currently under research in clinical trials, and some of them have already been approved for clinical application, including stem cell therapy, targeted therapy, ablation therapy, photodynamic therapy (PDT), photothermal therapy (PTT), chemodynamic therapy (CDT), sonodynamic therapy (SDT), and ferroptosis-based therapy (FBT) (Debela et al., 2021).

**FIGURE 1:**
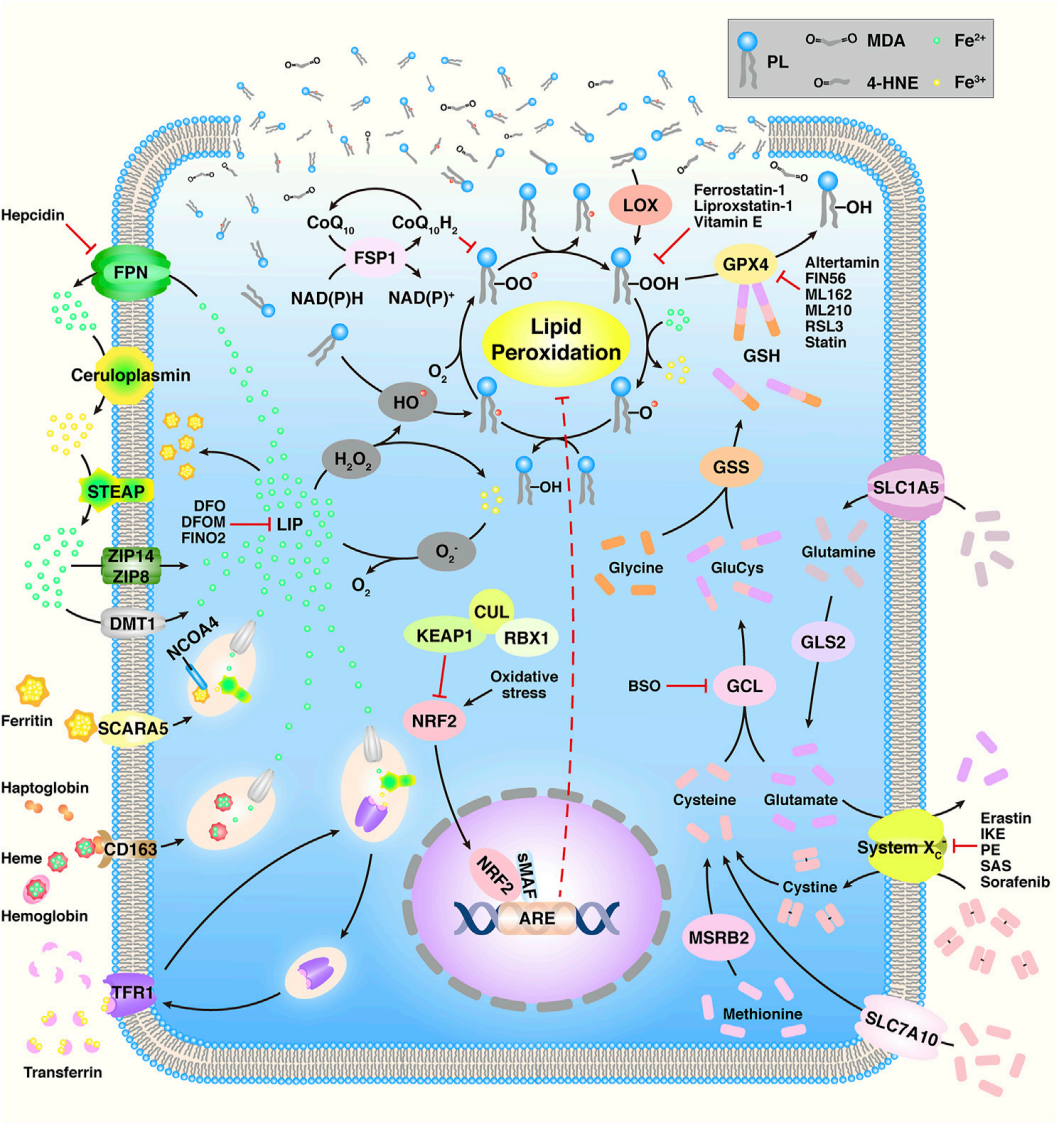
Overview of the ferroptosis mechanism. Adapted from Shi et al., (2021) Copyright @ 2021 (Frontiers).

Ferroptosis is a novel type of regulated cell death (RCD) that is iron-dependent and characterized by the oxidization of polyunsaturated fatty acids (PUFAs) and subsequently accumulation of lipid peroxides (LPOs) (Figure 1) (Shi et al., 2021). During the past two decades, Brent R. Stockwell et al. focused on ferroptosis, and their research studies have attracted global attention. Specifically, in 2003, they identified a novel compound and named it “erastin” (Dolma et al., 2003). Intriguingly, they found that although cell death occurred, characteristics of apoptosis were not observed when erastin was present in several tumor cells. In 2007, they found that erastin-induced cell death could be suppressed by α-tocopherol (Yagoda et al., 2007). In 2008, they found another small compound, Ras selective lethal 3 (RSL3) that induced analogous iron-dependent nonapoptotic cell death in RAS-enriched cancer cells, and this could also be restrained by the presence of α-tocopherol or desferrioxamine mesylate (DFOM) (Yang and Stockwell, 2008). In 2011, they confirmed that erastin- and RSL3-induced cell death differed from the mechanism of other cell deaths (Wolpaw et al., 2011). Therefore, in 2012, they named this phenomenon ‘ferroptosis’ (Dixon et al., 2012). Ferroptosis is distinct from apoptosis, autophagy, and necroptosis in terms of morphology, biochemistry, and genetics (Cao and Dixon, 2016). Cells experiencing ferroptosis show special hallmarks, including the fracture of the cell membrane, smaller mitochondria, increased density of the mitochondrial membrane, reduced/vanished mitochondrial cristae, and outer mitochondrial membrane breakup; however, the nuclei remain normal (Li, J. et al., 2020).

Many nanotechnologists have focused on cancer therapy in recent years because of the promising physicochemical properties of nanomaterials (Liang et al., 2019), and an increasing number of biomedical nanomaterials have been widely investigated as nanomaterial-based drug delivery systems for anticancer therapeutics, with enhanced drug availability, improved efficacy, targeted delivery, and reduced treatment-related toxicity, which could better solve the disadvantages of anticancer drugs with low solubility and poor membrane permeability (Luo et al., 2014; Asghari et al., 2019; Fei et al., 2020a). Nanomaterials possess an exceptional advantage of the enhanced permeability and retention (EPR) effect, which is related to anatomical and pathophysiological differences between the tumor tissue and normal tissue. Crucially, the solid tumors are highly vascularized and have large gaps between the endothelial cells in tumor vessels; as a result, macromolecular drugs are selectively extravasated and retained by the tumor tissues (Fang et al., 2011). Nevertheless, the EPR effect can be influenced by the systolic blood pressure and is negligible in advanced cancers (Maeda 2015; Golombek et al., 2018; Shan et al., 2020). In addition to the EPR effect, several strategies were taken into advantage by the researchers, such as modifying targeting molecules, remodeling the tumor microenvironment (TME), and enabling tumor stimuli–responsive properties (Shi et al., 2017; Youn and Bae, 2018; Park et al., 2019). Thus, nanomaterials can be used as delivery agents for anticancer drugs, but they can also be used to convert energy to kill cancer cells, such as light, ultrasound (US), electricity, and magnetothermal energy (Fei et al., 2020a). As a result, in the last few years, the Food and Drug Administration (FDA) has approved a large number of anticancer nanomaterials, demonstrating the huge potential of nanomaterials for the precision and personalized cancer therapies in future.

Ferroptosis, as a novel RCD, has become a popular area of cancer research, and, thus, numerous nanomaterials that induce ferroptosis have been developed for cancer therapy because of their potential antitumor properties. In this review, we try to summarize and discuss the multifunctional ferroptosis-based nanomaterials on the recent advances and breakthroughs in cancer therapies.

## 2 FERROPTOSIS INDUCERS (FINs)

**FIGURE 2:**
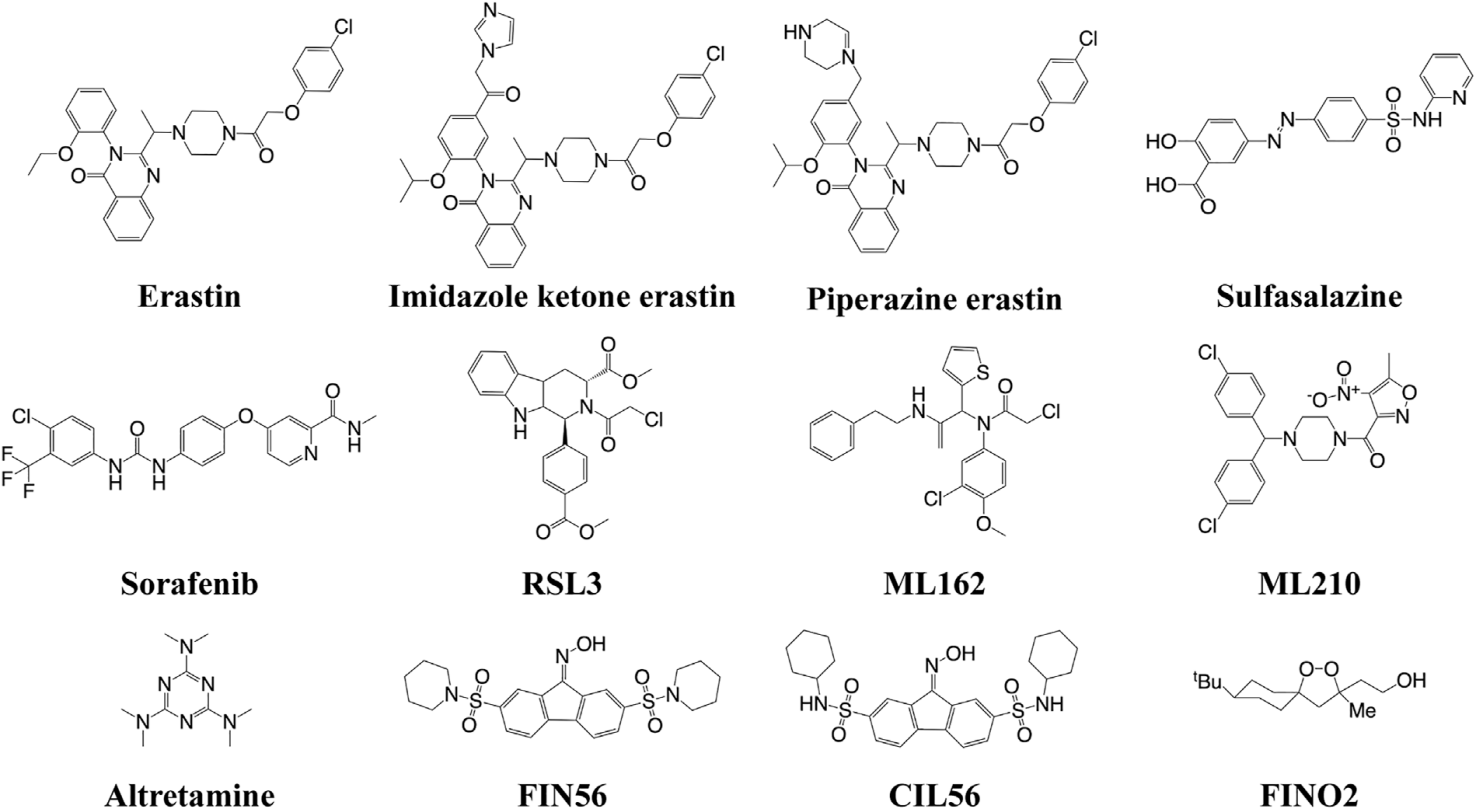
Structures of the small-molecule ferroptosis inducers.

The FINs can be classified into four types **(** Figure 2 **)**. Class I FINs could block the biosynthesis of glutathione (GSH) or deplete it. Many small compounds (such as erastin, imidazole ketone erastin, piperazine erastin, sulfasalazine, and sorafenib) can inhibit system X_c_^−^, which could transfer cystine into the cells and export glutamate out of the cells (Feng and Stockwell, 2018). The system X_c_^−^ consists of solute carrier family 7 member A11 (SLC7A11, also known as xCT) and SLC3A2 (4F2hc) (Shi et al., 2021). GSH is a cofactor of GSH peroxidase 4 (GPX4), which is a crucial regulator for the conversion of LPOs to the corresponding hydroxyl compounds (LOH). Therefore, the depletion of GSH can inactivate GPX4, subsequently resulting in the accumulation of LPOs to induce ferroptosis. Class II FINs directly inhibit GPX4. Small compounds (such as RSL3, ML162, ML210, and altretamine) can interact with the active selenocyteine site of GPX4 and inhibit its enzymatic activity, resulting in the accumulation of LPOs (Cao and Dixon 2016). Class III FINs deplete the GPX4 protein. For example, the compounds FIN56 and caspase-independent lethal 56 (CIL56) could induce ferroptosis by directly degrading the GPX4 and synchronously depleting the mevalonate-derived coenzyme Q_10_ (CoQ_10_) (Shimada et al., 2016). Class IV FINs could induce lipid peroxidation, but there is only one small compound, FIN endoperoxide (FINO2), that can directly oxidize iron, indirectly inactivate GPX4, and ultimately cause lipid peroxidation (Gaschler et al., 2018). This compound is suitable *in vitro*; however, it might not be effective *in vivo*.

## 3 Nanomaterials as FINs

**FIGURE 3:**
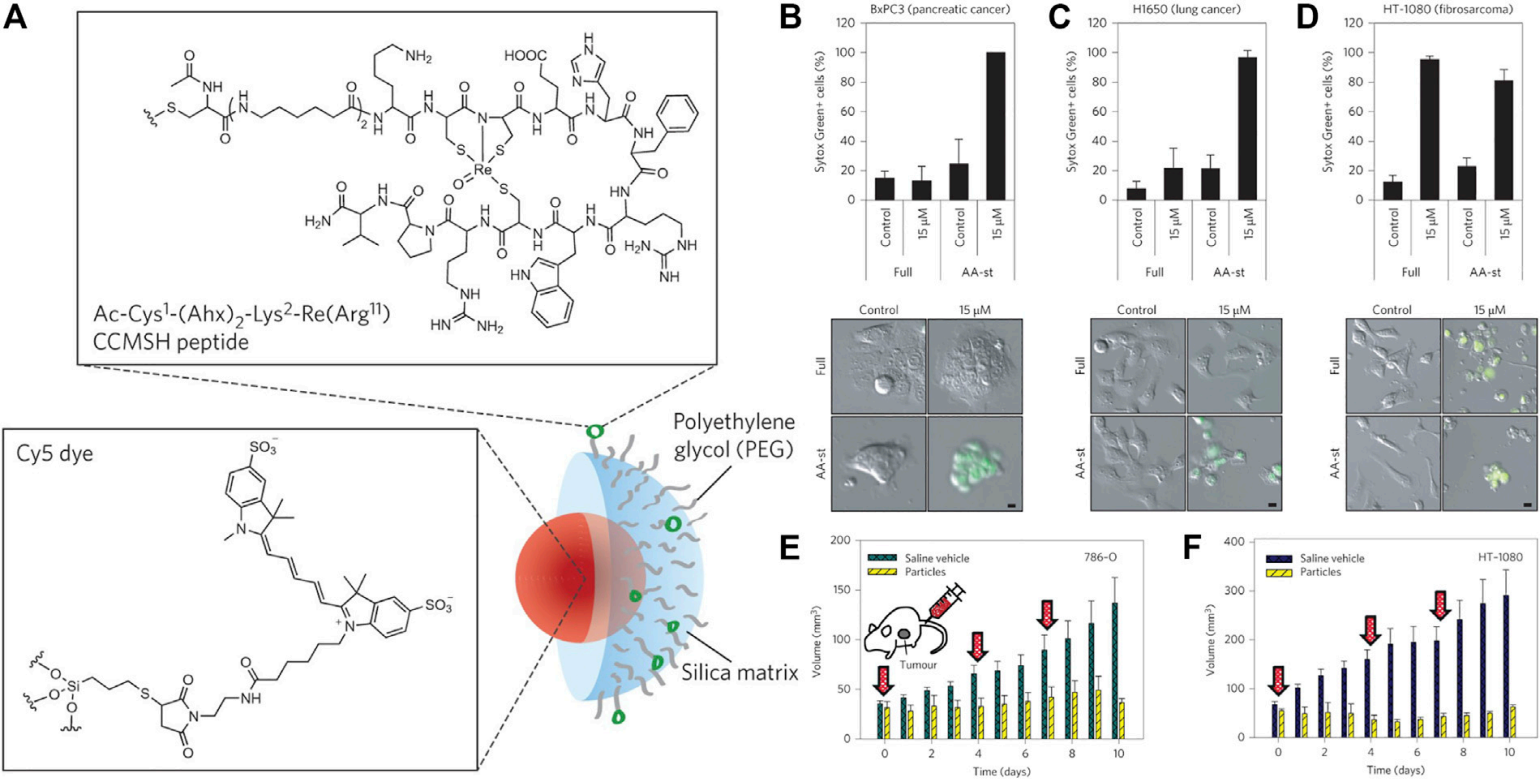
**(A)** Synthesis scheme of αMSH-PEG-C′ dots. **(B–D)** Cell death about BxPC3 cells with different treatments **(B)**, H1650 cells **(C)**, and HT-1080 cells **(D)**. **(E)** 786-O and **(F)** HT-1080 tumor volume with different treatments. Reproduced with permission from Kim et al., (2016) Copyright @ 2016 (Springer Nature).

The design of the multifunctional nanomaterials with precisely tuned physicochemical properties could enhance the drug delivery for cancer therapy. The first ferroptosis-inducing nanomaterial was reported by Kim et al. in 2016 (Figure 3) (Kim et al., 2016). They coated the near-infrared (NIR) fluorescent silica nanoparticles (NPs) with ploy (ethylene glycol)-coated (PEGylated) and Cornell dots (C′ dots) and surface-modified with the melanoma-targeting peptide and alpha-melanocyte stimulating hormone (αMSH). The C′ dots could absorb and incorporate extracellular iron into their structure to increase the intracellular iron level with the subsequent production of reactive oxygen species (ROS) and depletion of GSH, ultimately leading to ferroptosis in different cancer models. The research laid the groundwork for investigating nanomaterials as effective FINs in sensitive tumors.

### 3.1 Iron-Contained Nanomaterials

Owing to the crucial role of iron in ferroptosis, iron-contained nanomaterials have become the most popular nanomaterials for FBT. In recent years, several types of iron-contained nanomaterials have been explored to induce ferroptosis.

#### 3.1.1 Iron Ion–Based Nanomaterials

**FIGURE 4:**
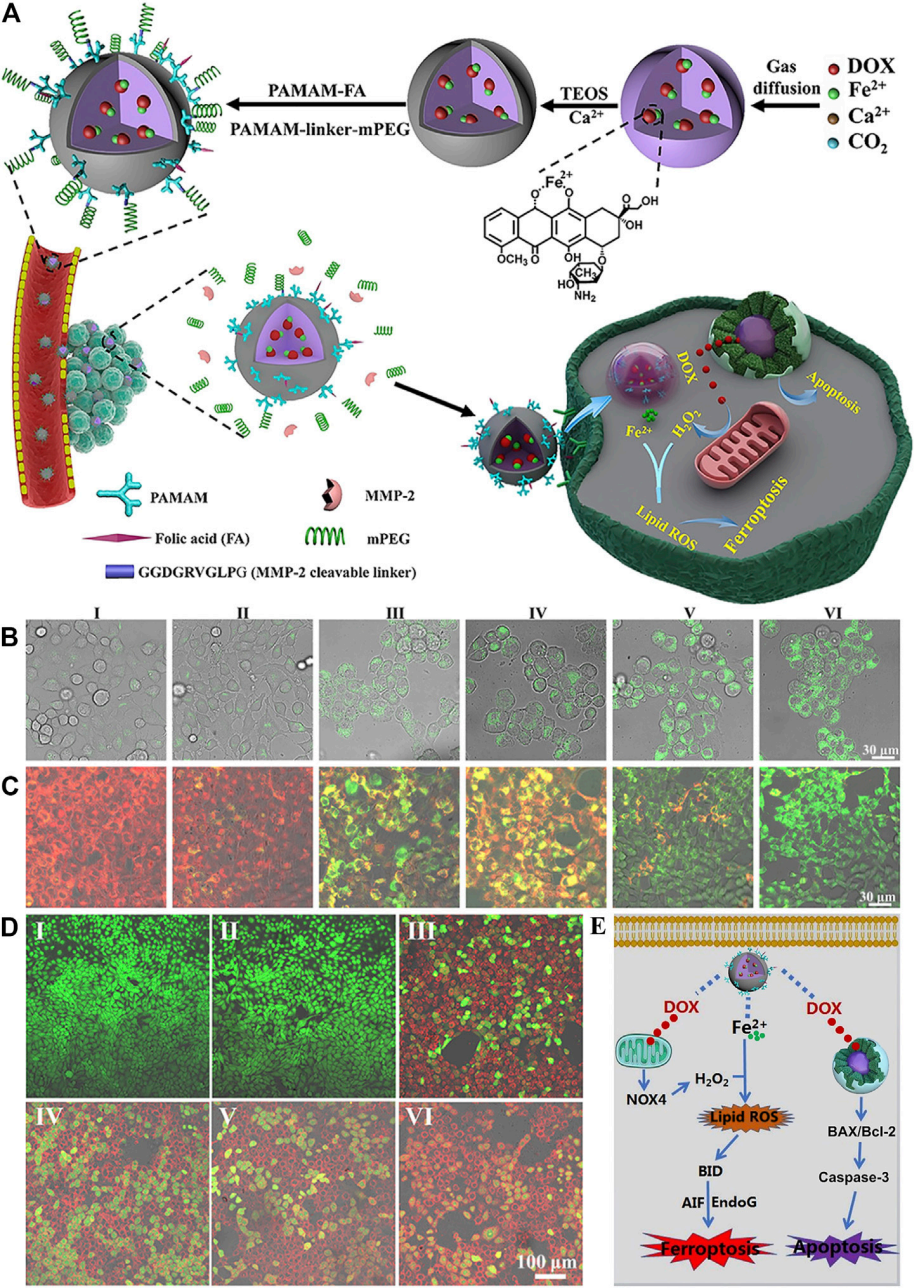
**(A)** Synthesis scheme of ACC@DOX-Fe^2+^-Ca-FA/mPEG and its therapeutic action. **(B–D)** Confocal laser scanning microscopy observation in 4T1 tumor cells about the intracellular distribution of the LPOs **(B)**, the changes in the mitochondrial membrane potential **(C)** and apoptosis levels **(D)** after treatment with (I) PBS, (II) ACC-Ca-FA/mPEG, (III) DOX, (IV) ACC@DOX-Ca-FA/mPEG, (V) ACC@DOX-Fe^2+^-Ca-FA/mPEG, and (VI) MMP-2–treated ACC@DOX-Fe^2+^-Ca-FA/mPEG. **(E)** Molecular mechanism for ACC@DOX-Fe^2+^-Ca-FA/mPEG-induced ferroptosis/apoptosis. Reproduced with permission from Xue et al., (2020) Copyright @ 2020 (AAAS).

The different valence states of iron have been exploited to develop nanomaterials that act as FINs to kill cancer cells. In the zero-valent iron NP (ZVI NPs)–sensitive cancer cells, Fe^0^ could be oxidized to Fe^2+^ (Huang et al., 2019). The increased iron induced intracellular ROS surge *via* Fenton reaction, followed by the accumulation of mitochondrial LPOs. The monodispersed ferrihydrite NPs were synthesized and demonstrated the light-triggered Fe^2+^ generation at the tumor sites (Yang, Y. et al., 2021). Ferrihydrite NPs, 20∼30 nm, could release a large amount of Fe^2+^ to promote the iron/ROS-related irreversible DNA fragmentation and GPX4 inhibition under a common blue light illumination. In addition, it simultaneously induced polarization of the tumor-associated macrophage (TAM) from the tumor-promoting M2 phenotype to the tumor-killing M1 type, which concomitantly inhibited the tumor growth and prevented lung metastasis under illumination *in vivo*. The sulfasalazine and disulfide-bridged levodopa (DSSD) assembled as sulfasalazine@DSSD that could chelate Fe^2+^ to form the prodrug nanosystem sulfasalazine–Fe^2+^@DSSD (Xin et al., 2021). The disulfide bonds could consume GSH in tumor cells, triggering the leakage of sulfasalazine and Fe^2+^. Fe^2+^ induced ferroptosis *via* the Fenton reaction, and this was enhanced by the presence of sulfasalazine to improve the therapeutic efficacy. In another system, doxorubicin (DOX) was chelated with Fe^2+^, yielding Fe^2+^-DOX, which was condensed with calcium-containing precursors to form amorphous calcium carbonate (ACC)–encapsulated Fe^2+^-DOX cores *via* a one-step synthetic approach (Figure 4) (Xue et al., 2020). The nanoplatform was simultaneously modified with matrix metalloproteinase-2 (MMP-2)–sheddable PEG and folic acid (FA) to achieve circulation stability and targeting capability. The therapeutic nanomaterial could release Fe^2+^ that produced H_2_O_2_ and DOX that amplified the effect of Fe^2+^ under a pH-triggered and self-regulated manner.

Recently, Cui et al. prepared a nanoenzyme in which Fe^3+^ was substantially reserved in the polypyrrole NPs (FePPy NPs) (Cui et al., 2021). The FePPy NPs generated hydroxyl radicals (•OH) by the degradation of H_2_O_2_ to induce ferroptosis. Upon irradiation, the FePPy NPs induced low-temperature PTT, which accelerated the Fenton reaction and enhanced ferroptosis and photoacoustic imaging (PAI) *in vitro* and *in vivo*. In addition, nanomaterials based on Fe^3+^ and tannic acid (TA) with the DOX-loaded dendrimers could combat multidrug resistance (MDR) in cancer cells *via* the apoptosis/ferroptosis pathway (Guo et al., 2019). The DOX-induced apoptosis elevated the intracellular ROS levels, thus sensitizing cancer cells to ferroptosis. The efficiency of CDT, in which H_2_O_2_ is decomposed into toxic •OH in tumor cells, was improved. Chen H. et al. reported a nanodrug delivery system based on TA, Pluronic F-68, and Fe^3+^ (Chen, H. et al., 2021). DOX, Pluronic F-68, and TA were first assembled to form DOX@Pluronic F-68/TA by π–π interactions and hydrophobic bonds. Subsequently, Fe^3+^ was introduced onto the surface of DOX@Pluronic F-68/TA to form DOX@Pluronic F-68/TA/Fe^3+^ *via* elaborate coordination interactions. The nanodrug DOX@Pluronic F-68/TA/Fe^3+^ efficiently targeted the tumor and primely suppressed tumor growth. In addition, the hollow mesoporous silica NP (MSNP)–loaded ferrate and DOX were followed by incorporating n-heneicosane (Fu et al., 2021). The mild hyperthermia induced by US initiated the phase change of n-heneicosane and enabled the co-release of ferrate and DOX. The ferrate then reacted with H_2_O to form H_2_O_2_, which released O_2_, thus causing TME-independent reoxygenation and downregulation of the expression of hypoxia-inducible factor 1α and MDR gene/transporter P-glycoprotein in tumor cells. Overall, the cooperation of chemotherapy and FBT resulted in enhanced suppression of hypoxic osteosarcoma growth *in vivo*.

**FIGURE 5:**
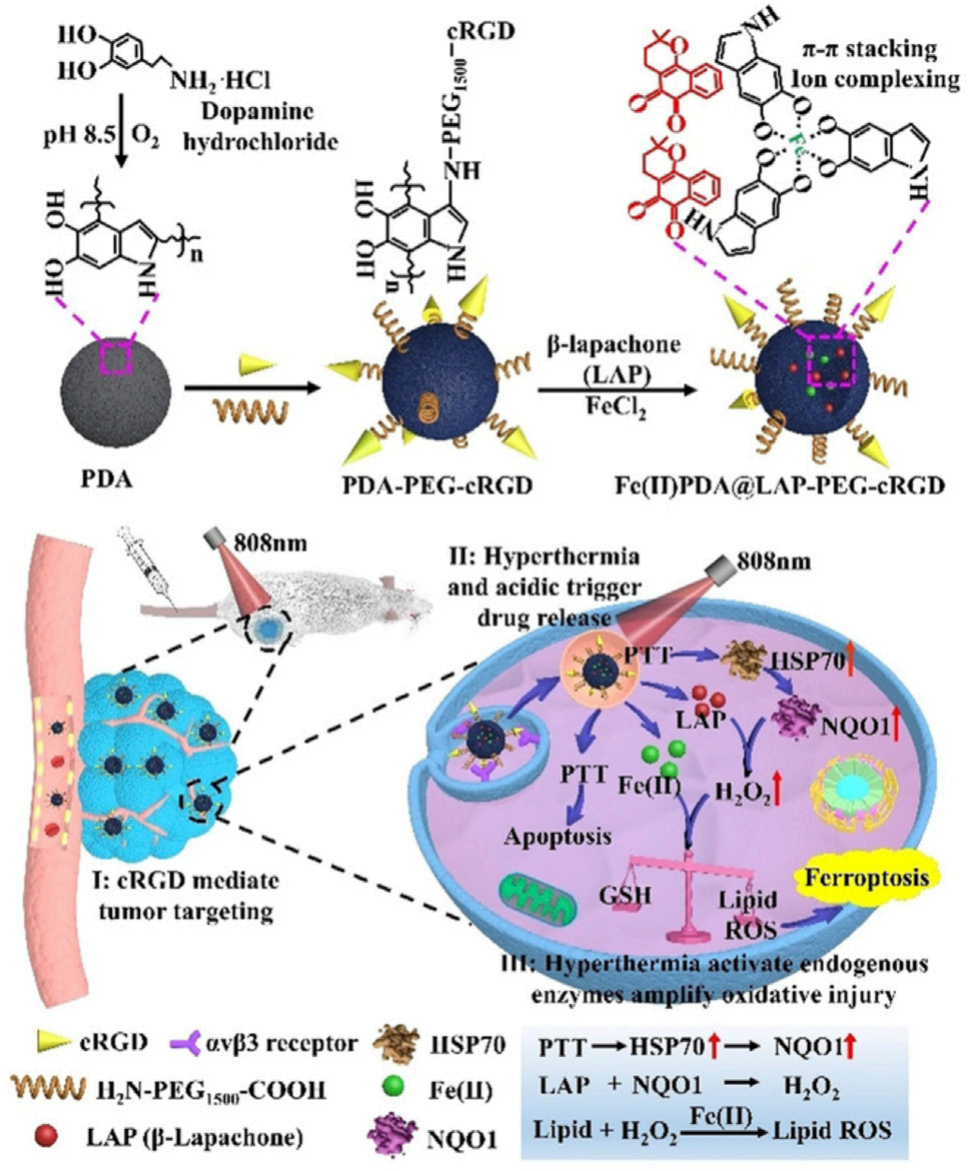
Synthesis scheme of the Fe^2+^PDA@LAP-PEG-cRGD nanomaterial and mechanism of PTT/FBT for cancer. Adapted from Xue et al., (2021) Copyright @ 2021 (Wiley).

Dopamine can be used to load hydrophobic drugs and metal ions due to its aromatic rings and catechol groups (Cheng et al., 2019). Furthermore, polydopamine (PDA) shows strong absorption in the NIR region at approximately 808 nm and can efficiently convert the light energy into thermal energy (Rahim et al., 2019;Yamagishi et al., 2019). PDA nanosubstrates could efficiently load Fe^2+^ and β-lapachone, which initiated ferroptosis in the tumor cells subjected to NIR illumination (Xue et al., 2021). Crucially, cellular hyperthermia was generated by PDA nanostructures under NIR irradiation and triggered the release of Fe^2+^. The NIR-enabled PTT also activated heat shock response and upregulated NADPH: quinone oxidoreductase protein (NQO1) *via* the heat shock protein 70 (HSP70)/NQO1 axis to promote bioreduction of β-lapachone and boost the intracellular H_2_O_2_ levels to facilitate the Fe^2+^-dependent lipid peroxidation, while minimizing the potential damage to normal tissues (Figure 5) (Xue et al., 2021). The Fe^2+^ and Fe^3+^ also could be loaded into the core of the ultrasmall PEG-modified PDA NPs (UPDA-PEG NPs, 8.4 nm), thus yielding a novel ferroptosis agent (Chen et al., 2019). Seventy percent of the iron ions could be released at pH 5.0, suggesting applications in the acidic TME. The ferroptosis induced by UPDA-PEG@Fe^2+^ NPs was dependent on ROS, whereas UPDA-PEG@Fe^3+^ NPs induced LPO-dependent ferroptosis. In another research, DOX was loaded onto the PDA NPs to achieve a synergistic effect (Nieto et al., 2021). The pH determined the Fe^3+^ state and the selectivity and therapeutic activity of the resultant Fe^3+^-loaded PDA NPs. Furthermore, the antitumor activity could be enhanced by DOX, which also increased ROS production in the cancer cells, and PDA NPs with different concentrations of Fe^3+^ and drugs could be tailor-synthesized and administered depending on the therapeutic need. The Fe^3+^ and the catechol of dopamine molecules could self-polymerize Fe^3+^-PDA cores with an Fe loading efficiency of 5.3% (Chen et al., 2020). Subsequently, the hyaluronic acid (HA) cross-linked cisplatin shells were constructed onto the Fe^3+^-PDA cores, and the core-shell nanomaterial was highly sensitive to an acidic or reductive TME, while its disassembly led to the continuous release of cisplatin and Fe^3+^. The cisplatin could enhance ferroptosis *via* activating the NADPH oxidases (NOXs) and suppressing GPX4. Thus, the core-shell nanomaterial provided a new antitumor strategy for synergistic chemotherapy/FBT/PTT with a low dosage.

Iron ions could also be coordinated to some nanomaterials. For example, Jiang Y. et al. reported a hybrid semiconducting nanozyme (HSN) possessing high photothermal (PT) conversion efficiency and NIR-II PAI-guided PTT/FBT (Jiang, Y. et al., 2020). HSN comprised the amphiphilic semiconducting polymer as a PT converter, PA emitter, and iron-chelating Fenton catalyst. Upon NIR-II irradiation, HSN induced PT transduction, which triggered PTT and potentiated the Fenton reaction. Furthermore, •OH transformed HSN to approximately 1.7 nm–sized fragments. The noninvasive therapy possesses several advantages, such as deep ablation, reduced expression of metastasis-related proteins, and inhibition of distant metastasis. Recently, artemisinin and its derivatives have been investigated as potential FINs for cancer treatment. The nanocarrier-containing artemisinin was based on TA and Fe^2+^ coated on the zeolitic imidazolate framework-8 (ZIF-8) (Li, Z. et al., 2021). After 10 h, 59% artemisinin was released from TA-Fe^2+^/artemisinin@ZIF-8 in pH 5.0. The intracellular ROS production and GPX4 inhibition could lead to suppression of triple-negative breast cancer growth.

#### 3.1.2 Iron‐Based Metal‐Organic Frameworks (MOFs)

The nanoscale MOFs formed by Fe^2+^ and 2-aminoterephthalic acid (BDC-NH_2_) possessed excellent stability and pH-responsive degradation to release Fe^2+^ in the acidic TME, which could catalyze the Fenton reaction and produce considerable quantities of ROS to induce Fe^2+^-mediated ferroptosis (Xu et al., 2020). The perfluoropentane@Fe/Cu-SS MOF is displayed as an effective FIN because it could increase the production of LPO through the Fenton reaction and inhibit the GPX4 that prevents LPO transfer to LOH in the presence of GSH, whereas the Fe^3+^ and Cu^2+^ in the MOF could consume GSH, further inhibiting GPX4 activity. Perfluoropentane@Fe/Cu-SS MOF also enabled T1-weighted magnetic resonance imaging (MRI), US imaging, and PTT (He, H. et al., 2020). Au1Ag24 nanoclusters and Fe^3+^ could be effortlessly assembled in an oil phase and modified by the vesicles of the PEG block grafted polyketal copolymer (PbP). The core-shell cancer theranostic nanomaterial Fe^3+^@Au1Ag24@PbP possessed several excellent multiproperties, including NIR laser/TME co-responsiveness and PAI/PT imaging-guided PTT/PDT/FBT both *in vitro* and *in vivo* (Cheng, H. et al., 2021). In addition, the iron-chelated hybrid polymeric NPs comprising Fe^3+^ and an amphiphilic semiconducting complex have been reported, in which these components acted as PT transducers and Fe^3+^ chelators, respectively (He, S. et al., 2020). In the acidic TME, Fe^3+^ was released to generate •OH; in addition, local heat was generated under the NIR laser irradiation to enhance the Fenton reaction and achieve PAI-guided PTT.

**FIGURE 6:**
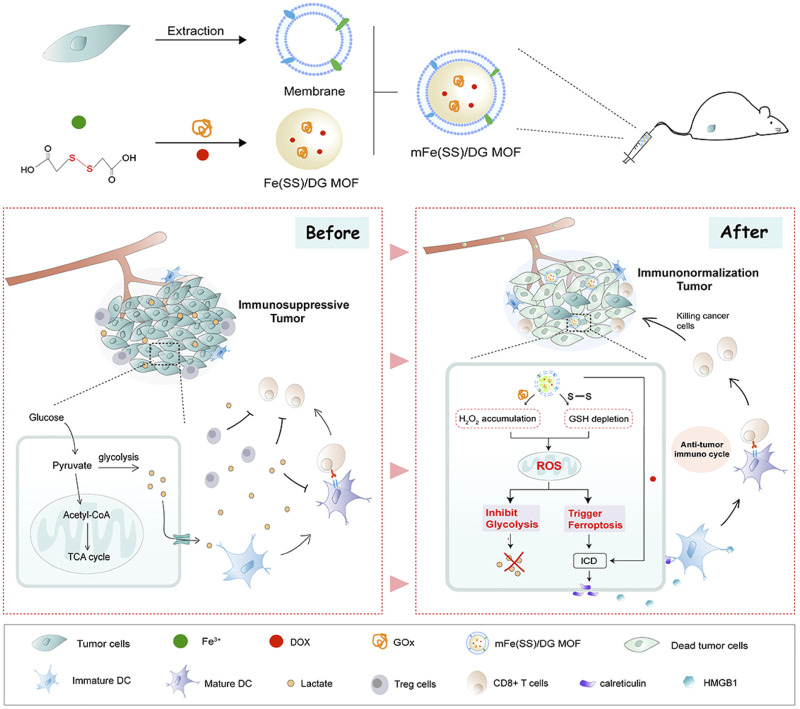
Schematic illustration of mFe (SS)/DG and the mechanism based on ferroptosis–glycolysis regulation. Adapted from Yang J et al., (2021); Yang Y et al., (2021) Copyright @ 2021 (Elsevier).

The addition of cancer cell membranes to nanomaterials enables homologous targeting and immune escaping capabilities. Wan et al. reported a nanomaterial containing Fe^3+^-based MIL-100 and glucose oxidase (GOx) that catalyzed the degradation of glucose to generate sufficient H_2_O_2_ for FBT (Wan et al., 2020). When the nanomaterial was obtained by the tumor cells, intracellular GSH triggered the disruption of MOF *via* reducing Fe^3+^ to release Fe^2+^ and GOx. The nanomaterial exhibited highly effective tumor inhibition and enabled precise collaborative tumor therapy with spatiotemporal controllability. Another MOF nanomaterial loading with GOx and DOX was also coated with the cancer cell membrane (denoted as mFe(SS)/DG) (Figure 6) (Yang, J. et al., 2021). The mFe(SS)/DG system based on Fe^3+^ and disulfide-bearing ligands depleted GSH and suppressed GPX4 activity to induce ferroptosis. The increasing ROS facilitated ferroptosis and restrained glycolysis. Thus, immunogenic cell death (ICD) induced by the synergistic effect of ferroptosis and DOX released tumor antigens to trigger antitumor immunity when the inhibition of glycolysis remodeled the tumor immunogenicity and immunosuppressive microenvironment to strengthen the antitumor immunity.

In addition, iron-based MOFs can be loaded with FINs to enhance ferroptosis. For example, MIL88B loaded with RSL3 forced M2 macrophages to increase the glycolytic metabolism (Gu et al., 2021). MIL88B/RSL3 downregulated the expression of M2-associated signaling pathways and activated M1-related signaling. The ferroptosis-strengthened macrophage regulation strategy had great potential for TAM-centered antitumoral applications. In the same year, the surface of MIL-101 (Fe)-loaded sorafenib was modified with an iRGD peptide that could bind to neruopilin-1 receptor yielding a nanodrug that induced ferroptosis of the hepatocellular carcinoma by consuming GSH, decreasing GPX4 expression, and enhancing LPO generation (Liu et al., 2021). Fe^3+^ and TA spontaneously formed a network-like corona onto the sorafenib nanocores that were loaded with methylene blue (Liu et al., 2018). The corona is disrupted in the lysosomal acid environment, whereas TA could reduce Fe^3+^ to Fe^2+^, paving the way for iron redox cycling to produce LPO. Furthermore, the concomitant methylene blue release induced fluorescence recovery, endowing the nanomaterials with multimodal imaging-guided PDT/FBT. In another example, Fe^3+^-containing MOFs loaded with piperlongumine (a FIN) were coated with a transferrin-decorated pH sensitive lipid layer to form the nanodrug (Xu, R. et al., 2021). Crucially, after treatment with this nanoagent, the cellular iron concentration increased dramatically due to the iron-containing MOF, and piperlongumine strengthened the ferroptosis *via* providing H_2_O_2_ for the dual induction system.

#### 3.1.3 FePt-Based Nanomaterials

FePt-based nanomaterials have potential clinical applications in cancer therapy. For example, a ferroptosis nanotheranostic agent was designed by integrating FePt as the core, Eu^3+^-based luminescent molecule, and FA as the targeting molecule (Yue et al., 2018). FePt-based nanomaterials exhibited an excellent MRI, computed tomography (CT), and time-gated luminescence properties in the clinical diagnosis and had excellent capacity to trigger ferroptosis of cancer *in vitro* and *in vivo*.

**FIGURE 7:**
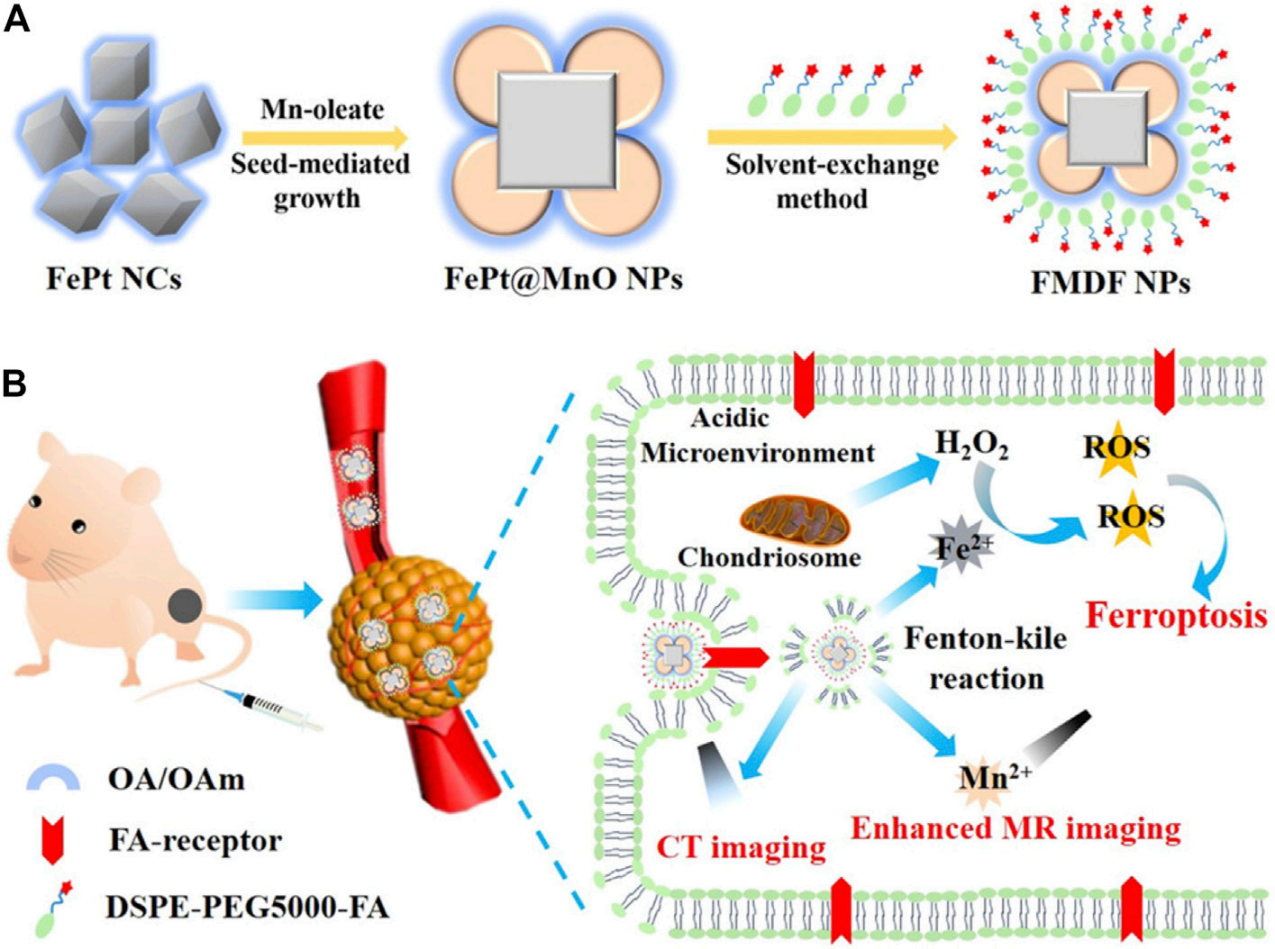
**(A)** Synthesis scheme of FMDF NPs. **(B)** Acidic-responsive and dual-model imaging-guided FBT/CDT. Adapted from Yang et al., (2019) Copyright @ 2019 (ACS).

The FePt NPs can also be loaded onto diverse nanomaterials to show their advantages. An acidic responsive nanoagent FePt@MnO@DSPE-PEG-FA NP was successfully constructed for MRI-guided FBT/CDT of cancer (Figure 7) (Yang et al., 2019). It could release active Fe^2+^ to elevate ROS production *via* the Fenton reaction to induce ferroptosis. In addition, Mn^2+^ could be released from the nanomaterial in the acidic TME and scavenge GSH to enhance the T1/T2-weighted MRI, which could obviously distinguish the solid tumors. Prussian blue (PB) nanocubes were utilized as PT agents, and FePt NPs were loaded onto the surface of the nanocube *via in situ* reduction (Hu et al., 2020). The surface of the nanocomposite PB@FePt was wrapped with HA and PEG, and the nanoagent PB@FePt–HA-g-PEG served as a multifunctional nanomaterial, enabling CDT/PTT and triple-modal imaging (MRI/CT/PT imaging) capability. Covalent organic polymers (COPs) possess specific surface area and good biocompatibility, making them promising nano delivery carriers (Shi et al., 2019). FePt@COP-FA with excellent PT potential was developed for cancer treatment (Meng et al., 2020). It could efficiently ablate the primary tumors with the presentation of NIR irradiation and release a mass of tumor-associated antigens *in situ*. With the assistance of anticytotoxic T-lymphocyte antigen-4 (anti-CTLA4), the specific immune response was triggered to suppress the growth of metastatic tumors. In particular, the synergistic therapy could form a valid immunological memory to further restrain tumor relapses. FePt NPs could also be loaded onto polyethylenimine (PEI)-modified ultrathin black phosphorus (BP) nanosheets, and this nanomaterial showed enhanced synergistic PTT/PDT/CDT for targeting primary tumors (Yao et al., 2020). More significantly, combined with the CTLA-4 checkpoint blockade, PTT induced by FePt/BP–PEI–FA could control the growth of both the primary and untreated distant tumor.

#### 3.1.4 Iron Oxide (IO)–Based Nanomaterials

IONPs, which have been approved by the FDA, have been widely applied in cancer therapy because they produce ROS *via* the Fenton reaction.

An innovative local chemotherapy combined with a gene therapy–based IONP was developed as a treatment for patients with glioblastoma (Zhang et al., 2020). The porous structure of IONPs attached -COOH groups and could encapsulate the small interfering RNA (siRNA) targeting GPX4 (siGPX4) and cisplatin. After uptake, the IONPs degraded and released Fe^2+^/Fe^3+^, and the H_2_O_2_ level increased owing to the activating reduced NOX. Furthermore, the cisplatin destroyed both nuclear and mitochondrial DNA, while siGPX4 restrained GPX4 expression. The manganese-deposited IO (FMO) NPs were loaded with the cisplatin prodrug Pt (IV) to form Pt-FMO, which was modified with PEG to increase colloidal stability (Cheng, J. et al., 2021). FMO released Mn^2+^ and Fe^2+^/Fe^3+^ in the weakly acidic TME, and the Pt (IV) could deplete GSH to generate cisplatin. Mn^2+^ enhanced the ferroptosis induced by Fe^2+^/Fe^3+^ released from FMO, and cisplatin elevated the intracellular H_2_O_2_ levels, which obviously strengthened ferroptosis *via* the Fenton reaction. Therefore, the synergism between the ferroptosis induced by FMO and the apoptosis induced by cisplatin resulted in tumor ablation.

**FIGURE 8:**
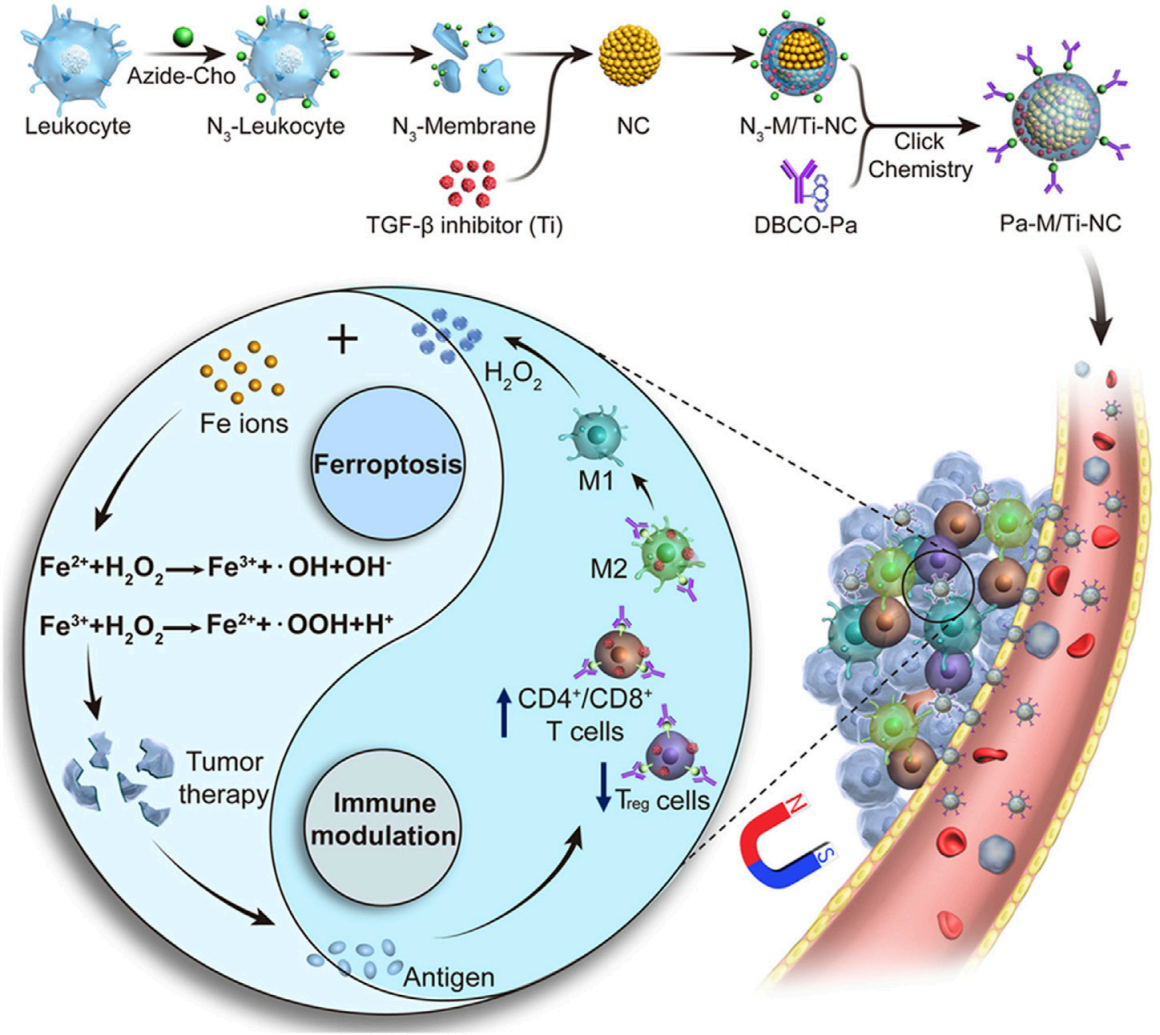
Synthesis scheme and mechanism of the magnetosome for FBT/immunomodulation in cancer. Adapted from Zhang et al., (2019) Copyright @ 2019 (ACS).

Biomimetic magnetic NPs coated with platelets can make the cancer cells sensitize to ferroptosis, generate mild immunogenicity, and improve the response rate of noninflammatory tumors to immunotherapy (Jiang, Q. et al., 2020). Thus, a Fe_3_O_4_-sulfasalazine@platelet system was designed by using mesoporous magnetic Fe_3_O_4_ NPs, sulfasalazine, and platelet membranes. Fe_3_O_4_–sulfasalazine@platelet-mediated ferroptosis could evidently improve the potency of programed cell death 1 (PD-1) and achieve persistent tumor elimination in 4T1 metastatic tumors. Moreover, the Fe_3_O_4_–sulfasalazine@platelet-mediated ferroptosis also repolarize macrophages from the M2 phenotype to M1 phenotype. The magnetosomes contained Fe_3_O_4_ magnetic nanoclusters and pre-engineered the leukocyte membranes as the core and shell, and the transforming growth factor-β (TGF-β) inhibitor and PD-1 antibody were, respectively, loaded inside and on the surface of the membrane (Figure 8) (Zhang et al., 2019). The membrane camouflage endowed the nanomaterial with a long circulation time, and the nanocluster core enabled magnetic targeting under MRI guidance. When the nanomaterial reached the tumor site, the cooperation of the TGF-β inhibitor and PD-1 antibody increased the rates of CD4^+^ T/Treg cells, CD8^+^ T/Treg cells, and M1/M2 macrophages to create an immunogenic TME, and the elevated H_2_O_2_ from M1 polarization enhanced the Fenton reaction. The production of •OH subsequently induced ferroptosis of the cancer cells and the released tumor antigens, thus increasing TME immunogenicity.

IONPs can also be loaded onto or into different nanomaterials. Superparamagnetic IONPs (SPIONPs) are considered excellent carriers to deliver nucleic acid and drug because of the controllable properties, outstanding stability, and easy modification. The sorafenib and ultrasmall SPIONPs (approximately 8.5 nm) were, respectively, loaded onto the mesopores of mesoporous PDA (MDPA) NPs and onto the surface of the MDPA NPs to form sorafenib@MPDA-SPIONP (Guan et al., 2020). The heat generated by the MPDA NPs upon NIR laser irradiation boosted the effect of ferroptosis. Furthermore, the application of moderate heat (45°C) in the presence of the IONPs could significantly suppress the expression of the antioxidative proteins and cause tumor cells to undergo specific lipid metabolism (Xie et al., 2021). By virtue of the IONPs and 1H-perfluoropentane (1H-PFP), a heat-responsive ferroptosis strategy was rationally designed by utilizing a polypeptide-modified, IO-containing nanomaterial that loaded 1H-PFP. The phase transition of 1H-PFP was triggered under 808-nm laser irradiation, which led to the release of IONPs *in situ* to produce ROS in the TME. The inhibition of GSH synthesis restrained the antioxidant response of tumors due to heat stress. Furthermore, the combined treatments reprogramed the lipid metabolism by generating a large amount of LPO and inducing acyl-CoA synthetase bubblegum family member 1 (ACSBG1)–dependent ferroptosis in the C4-2 tumor model. More recently, the polypeptide vehicle-based theranostics (Pt&IONPs@PP) were self-assembled by mixing poly (L-glutamic acid) (PGA), poly (L-lysine) (PLL), Pt prodrug, and carboxyl-modified IONPs through electrostatic interactions, and the surface of this nanomaterial was modified with PEG (Gao et al., 2020). The acidic TME degraded Pt&IONPs@PP to release Pt drug and Fe^2+^/Fe^3+^. Moreover, Pt&IONPs@PP could induce T2-weighted, MRI-guided chemotherapy/FBT to inhibit cancer cell growth without systemic toxicity *in vitro* and *in vivo*.

A nanosystem containing Fe_3_O_4_ and chlorin E6 (Ce6) was designed for the synergistic cancer therapy (Chen, Q. et al., 2021). The poly (lactic-co-glycolic acid) (PLGA) was used as the shell to load Fe_3_O_4_ and Ce6, which degraded in the acidic TME and released Fe^2+^/Fe^3+^ and Ce6. The reaction between Fe^2+^/Fe^3+^ and intracellular H_2_O_2_ produced •OH to induce ferroptosis of tumor cells. The released Ce6 could increase the ROS levels upon laser irradiation to offer PDT, and this boosted the ferroptosis effect in 4T1 tumors. A gel-delivery platform with embedded Au nanorods (AuNRs) and IONPs was reported, the so-called AuNRs&IONPs@Gel, for bladder cancer therapy (Guo, P. et al., 2020). The gel platform was based on a dextran aldehyde that could selectively adhere with the cancer collagen. AuNRs could induce imaging-guided PTT under NIR radiation, and the locally high concentration of IONPs induced ferroptosis. Moreover, TAMs would be repolarized by the IONPs from the M2 phenotype into M1 phenotype to achieve a direct antitumor effect and antigen presentation of dead cells. This process triggered an effective innate and adaptive immune response to protect against tumor rechallenge in the long term. The black hole quencher (BHQ)–based fluorescence ‘off−on’ nanomaterials were self-assembled with chitosan oligosaccharide (CSO), IR780, hexadecane (Hex), magnetic IONPs (MIONPs), and sorafenib to form CSO-BHQ-IR780-Hex/MIONPs/sorafenib (Sang et al., 2019a). BHQ and IR780 were combined *via* a GSH-responsive ether bond. After uptake, the CSO–BHQ–IR780–Hex NPs collapsed and IR780–Hex anchored the mitochondrial membrane. When NIR irradiation was applied to the NPs, the iron level increased, and the xCT/GSH/GPX4 system was triggered. In addition, a similar nanomaterial (CSO–SS–Cy7–Hex/SPIONP/sorafenib) was developed to overcome the therapy-resistant state of cancer (Sang et al., 2019b). This system could scavenge GSH and increase the concentration of iron and LPO, and the ferroptosis induced by CSO-SS-Cy7-Hex/SPIONP/sorafenib complex defeated MDR, reduced invasion, and limited the metastasis of cancers undergoing the epithelial-to-mesenchymal transition.

The IO-hydroxide (IOOH) nanospindles have been used as MRI contrast agents for cancer diagnosis; thus, a biocompatible H_2_S-responsive IOOH nanospindle for colon cancer is developed (Li, Y. et al., 2020). The IOOH nanospindles, synthesized from Fe^3+^ and dopamine and modified by PEG, could efficiently deplete endogenous H_2_S *via* the reduction reaction to inhibit CT26 colon cancer growth. Crucially, the overproduction of H_2_S drove a cascade reaction producing FeS; thus, this system could be used for NIR-responsive PTT and Fe^2+^-induced ferroptosis.

#### 3.1.5 Iron-Containing Organic Transition Metal Compound–Based Nanomaterials

**FIGURE 9:**
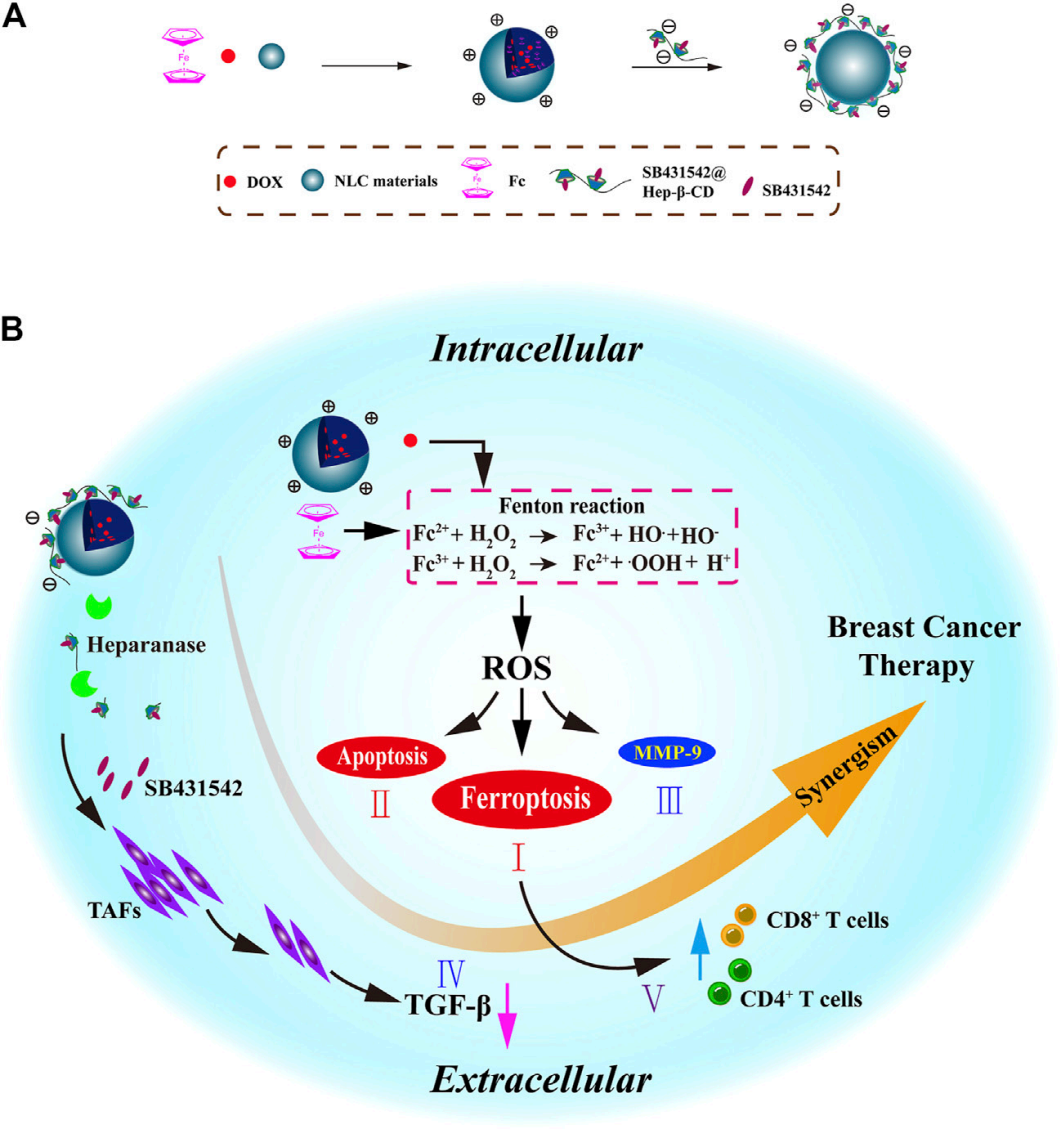
**(A)** Synthesis scheme of NLC/H (DOX + Fc + SB431542) NPs. **(B)** Mechanism of NLC/H (DOX + Fc + SB431542) NPs for breast cancer therapy. Adapted from Zhang et al., (2021) Copyright @ 2021 (Elsevier).

Ferrocene (Fc) is a stable organic transition metal compound that can produce ROS under physiological condition, and thus, is a potential adjuvant to enhance the therapeutic effect of chemotherapy drugs (Patra and Gasser, 2017). As known to all, the iron in Fc is Fe^2+^, (Xu et al., 2013) making it an ideal exogenous Fe^2+^ to induce ferroptosis. For example, sequential release of the NPs based on the presence of heparinase was reported; these NPs were modified with β-cyclodextrin and coloaded with DOX, Fc, and TGF-β receptor inhibitor SB431542 (Figure 9) (Zhang et al., 2021). DOX and Fc could increase the intracellular ROS levels to activate ferroptosis and apoptosis and reduce MMP-9 expression to enhance FBT in the tumor. SB431542 could be gradually released due to the heparanase-driven nature, which prevented the tumor metastasis *via* modulating the TME, decreasing tumor-associated fibroblast activation, and reducing TGF-β secretion. Furthermore, ferroptosis-induced antitumor immune response enhanced the tumor therapy. A CDT nanomaterial, RSL3@COF–Fc, was fabricated by the covalent organic framework (COF), Fc, and RSL3 (Zhou et al., 2021). The RSL3@COF–Fc could promote the production of •OH in cancer cells, and the repair mechanism under oxidative stress was attenuated by the irreversible inhibition of the GPX4. Finally, these two ways synergistically resulted in massive LPO accumulation.

A multifunctional carbon monoxide (CO)/thermo/chemotherapy nanoplatform comprises the mesoporous carbon NPs (MCNPs) as NIR-responsive drug carriers and DOX and hydrophobic iron carbonyl (FeCO) as thermosensitive CO prodrug (Yao et al., 2019). The nanoplatform produced sufficient heat upon NIR illumination to trigger the release of CO and DOX in the acidic TME. The CO successfully increased the sensitivity of the cancer cells to chemotherapeutics through the ferroptosis pathway. Subsequently, the FeCO-DOX@MCNP nanoplatform showed high therapeutic efficacy in combination with chemotherapy, PTT, and gas therapy both *in vitro* and *in vivo*.

#### 3.1.6 Ferritin-Based Nanomaterials

Ferritin is an iron-sequestering protein that plays key roles in iron delivery, cell proliferation, and immunosuppression (Shi et al., 2021). The novel protein-based nanomaterial BCFe@sorafenib was developed by covalently crosslinking the Ce6-conjugated bovine serum albumin (BSA) and ferritin, together with the sorafenib encapsulated inside the protein shell (Wang et al., 2021). Under hypoxic condition, the BCFe@sorafenib could be degraded to release more Ce6 after light irradiation, whereas the ferritin released Fe^3+^ to consume GSH to produce more ROS. In addition, the released sorafenib destroyed the tumor’s antioxidative defense to enhance oxidative damage. In addition, a novel carrier-free nanodrug NFER containing ferritin, erastin, and rapamycin was prepared by using an emulsification technique (Li et al., 2019). The NFER displayed strong ferroptosis capability by downregulating the GPX4 and increasing LPO production, which was increased by the presence of rapamycin. Using this nanodrug, tumor recurrence of the 4T1 tumor resection model was controlled.

#### 3.1.7 Iron‐Based Up‐Conversion (UC) Nanomaterials

**FIGURE 10:**
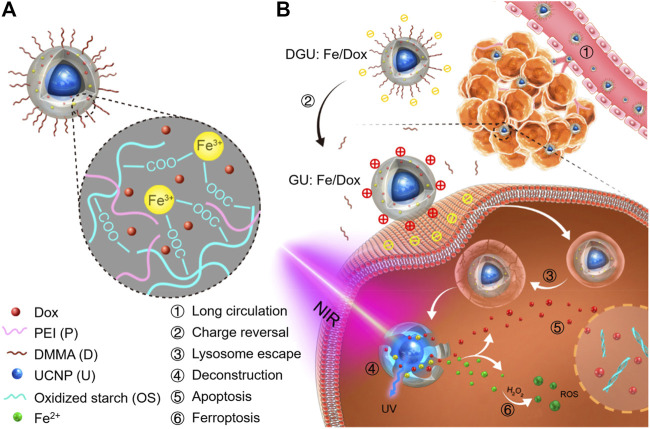
**(A)** Schematic illustration of DGU:Fe/DOX NPs. **(B)** Mechanism of DGU:Fe/DOX NPs for cancer therapy. Adapted from Bao et al., (2019) Copyright @ 2019 (ACS).

The photic UC is achieved by the UCNPs that contain lanthanide or actinide metals. The Fe^3+^-containing UCNP and DOX were capsulated into the oxidized starch-based gel NPs (Figure 10) (Bao et al., 2019). As the core, the UCNPs that convert NIR light to ultraviolet (UV) light overcome the obstacle of limited penetration depth and reduced Fe^3+^ to Fe^2+^, which led to the deconstruction of gel networks of this nanomaterial and subsequently rapid release of Fe^2+^ and DOX. As a result, this nanomaterial could provide a safe and efficient platform for ferroptosis-/apoptosis-based anticancer therapy.

### 3.2 Iron-Free Nanomaterials

In addition to the iron-contained nanomaterials, there are several types of nanomaterials without iron that could induce ferroptosis of the cancer cells.

#### 3.2.1 Manganese-Based Nanomaterials

**FIGURE 11:**
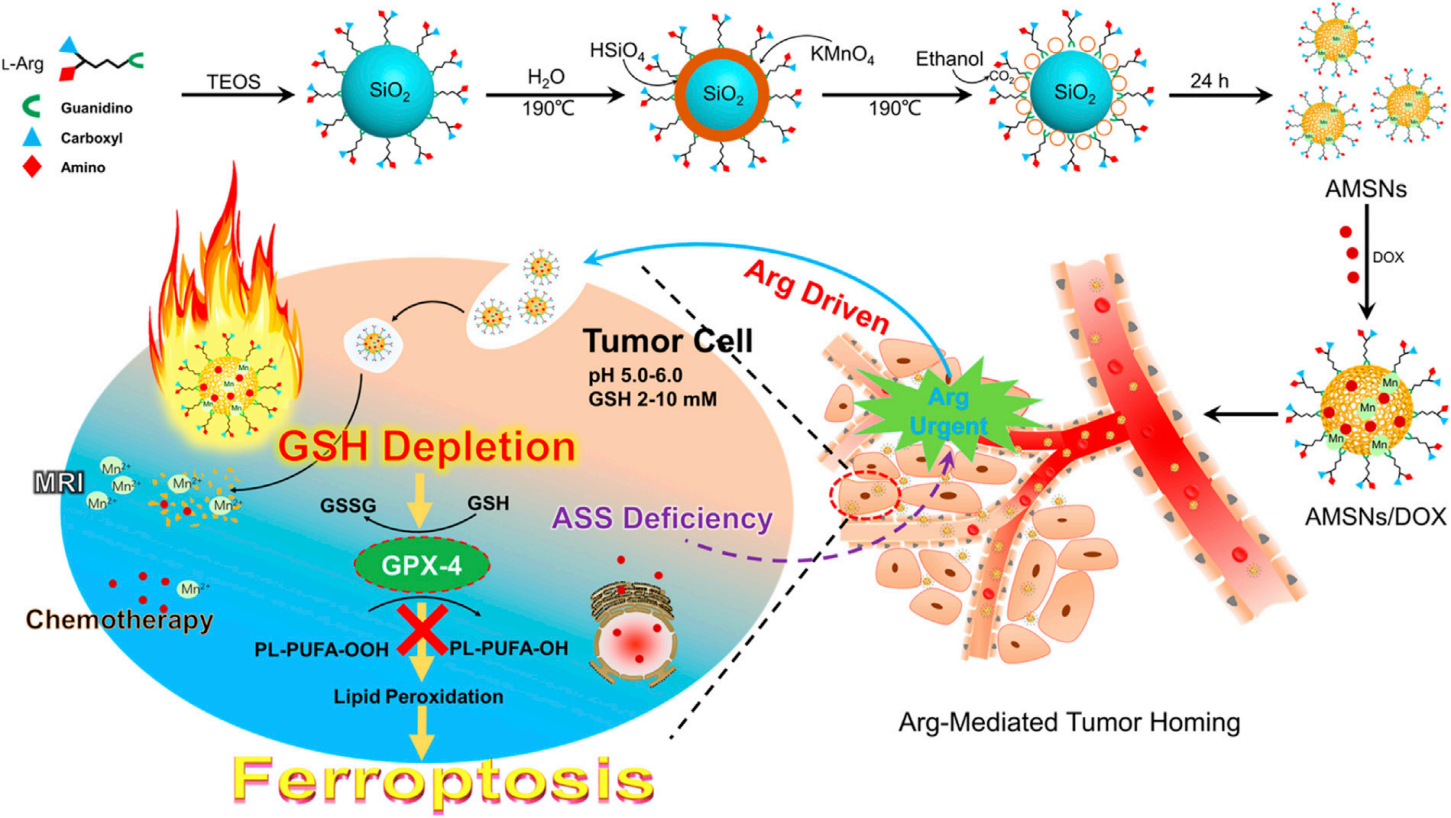
Schematic illustration of AMSNs/DOX for cancer therapy. Adapted from Wang et al., (2018) Copyright @ 2018 (ACS).

A versatile porphyrin-based manganese MOF (Mn-MOF) nanoplatform exhibited high catalase activity for the conversion of H_2_O_2_ to O_2_, thus decreasing the intracellular GSH content and GPX4 activity and inhibiting the tumor growth and metastasis *via* inducing SDT and FBT (Figure 11) (Xu, Q. et al., 2021). In addition, the Mn-MOF efficiently reshaped the tumor immune microenvironment upon US treatment by increasing the activated CD8^+^ T cells and matured dendritic cells (DCs) and decreasing the myeloid-derived suppressor cells in tumor tissue. The arginine-rich manganese silicate nanobubbles (AMSNs) could be synthesized through a one-pot method with highly efficient GSH depletion (Wang et al., 2018). The arginine component provides ideal dispersibility, biocompatibility, and tumor-targeting capacity. Compared with the traditional NPs, the arginine-based ultrathin surface coating and nanobubble structure significantly improved the ability of the AMSNs to consume GSH, which led to GPX4 inactivation. In addition, the degradation of AMSNs led to the corelease of Mn^2+^ and DOX, which resulted in T1-weighted MRI and on-demand chemotherapeutic drug release for the synergistic cancer therapy. In addition, manganese-doped MSNPs (MMSNPs) possess the manganese–oxygen bonds that could consume GSH rapidly (Tang et al., 2019). MMSNPs were modified with FA-PFG and loaded with dihydroartemisinin (DHA) to form an innovative nanomaterial (Fei et al., 2020b). After endocytosis, the degradation of nanomaterials consumed intracellular GSH due to the reaction between the manganese–oxygen bonds and GSH, which resulted in the release of DHA and Mn^2+^. The sorafenib could also be loaded into MMSNPs to induce ferroptosis of tumor cells (Tang et al., 2019; Tang et al., 2020).

#### 3.2.2 Manganese Oxide–Based Nanomaterials

**FIGURE 12:**
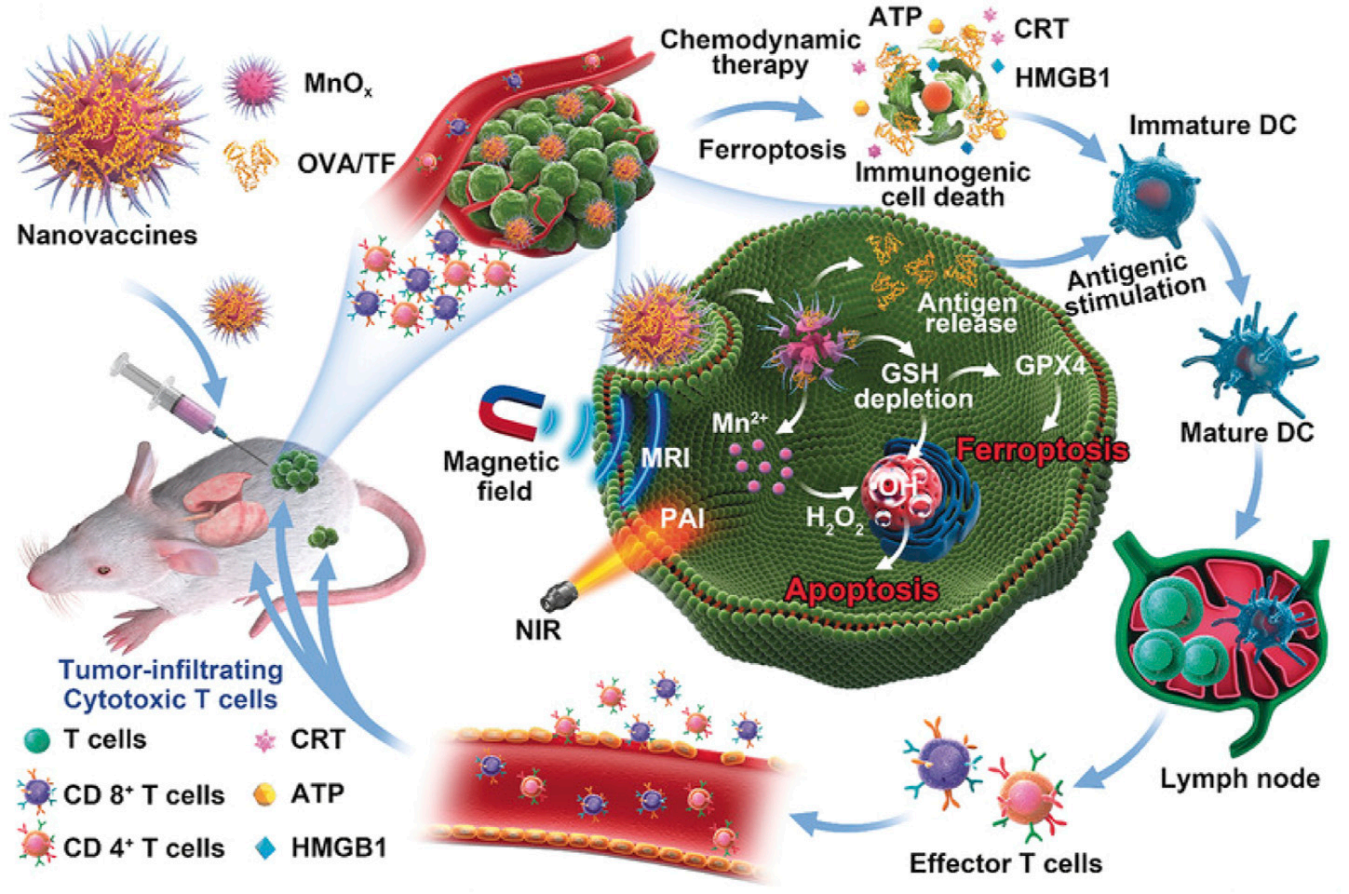
Schematic illustration of MnOx-OVA/tumor cell fragment (TF) nanovaccines for MRI-/PAI-induced cancer immunotherapy. Adapted from Ding et al., (2020) Copyright @ 2020 (ACS).

The MnO_2_@HMCu_2−x_S nanocomposites (HMCMs) for tumor ablation were reported (An et al., 2019). The HMCMs had the ability of depletion of GSH enhanced by PTT, thus inducing PTT-enhanced ferroptosis of cells by GPX4 inactivation. The GSH-responsive Mn^2+^ release increased the ROS concentration by a Fenton-like reaction, thus resulting in the accumulation of LPO. The rapamycin was encapsulated into the HMCM to improve sensitization of the tumor cells to ferroptosis. The HMCM showed an outstanding anticancer effect *in vitro* and *in vivo*. In addition, MnO_x_ nanospikes and ICD drugs were developed as cancer nanovaccines (Figure 12) (Ding et al., 2020). Because of the double induction of CDT mediated by Mn^2+^ and FBT that depleted GSH, MnO_x_ nanospikes could be used as an immune adjuvant for efficient antigen loading and ICD drugs. The nanovaccines achieved the TME-responsive, dual-mode MRI/PAI, while effectively inhibiting tumor growth and metastasis.

#### 3.2.3 Gold-Based Nanomaterials

The salinomycin that conjugated with the Au NPs was used to kill breast cancer stem cells that were derived from CD24^low^/CD44^high^ subpopulation (Zhao et al., 2019). The salinomycin-AuNPs exhibited higher ability to induce ferroptosis *via* higher iron accumulation and GPX4 inactivation. Pt-decorated Au nanostars were presented as novel nanoprodrugs for FBT against MDR tumors (Del Valle et al., 2020). Upon NIR light irradiation, Pt and Au were released, which resulted in GSH depletion, GPX4 inactivation, and accumulation of lipid hydroperoxides. The NIR light-activation of the prodrugs showed efficacy of FBT against tumors without long-term side effects *in vivo*.

#### 3.2.4 Photosensitizer-Based Nanomaterials

The Ce6 and erastin could be self-assembled to form a novel nanodrug *via* hydrogen bonding and π–π interactions (Zhu et al., 2019). The erastin could induce accumulation of LPO *via* the Fenton reaction. Under the light irradiation, the generation of O_2_ ensured efficient photochemical reactions to produce ROS. The imidazole ligand containing disulfide and Zn^2+^ was coordinated to form an MOF nanocarrier for Ce6 loading. Regardless of the light irradiation, nanocarriers loaded with Ce6 induced GSH depletion through the disulfide−thiol exchange reaction in 4T1 tumor cells (Meng et al., 2019). In the 4T1 tumor–bearing mouse model, the nanomaterials showed excellent *in vivo* tumor growth inhibition, and the animal survival rate was improved. However, the combined use of iron chelating agents weakened the antitumor ability of the nanocarrier because it restrained ferroptosis.

**FIGURE 13:**
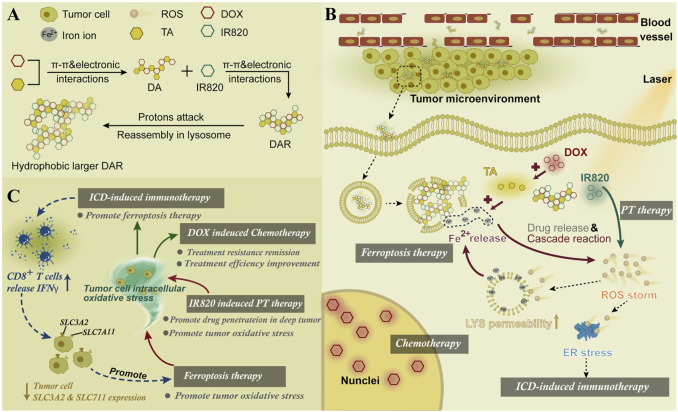
**(A)** Synthesis scheme of DAR. **(B)** Mechanism of DAR for cancer therapy. **(C)** Combined therapy mechanisms of DAR. Adapted from Xiong et al., (2021) Copyright @ 2021 (Elsevier).

The nanoactivator was assembled by DOX, TA, and IR820. The endogenous iron stored in the endo-lysosome was fully utilized. In addition, the ferroptosis and its related oxidative stress were induced by an artificial intracellular positive feedback loop (Figure 13) (Xiong et al., 2021). Interestingly, this process could also promote the ICD-related immunotherapy through the endoplasmic reticulum stress. After the laser treatment, the intracellular ROS in cells could be effectively distributed in the lysosomes and endoplasmic reticulum to promote FBT and immunotherapy, respectively.

#### 3.2.5 RSL3-Loaded Nanomaterials

**FIGURE 14:**
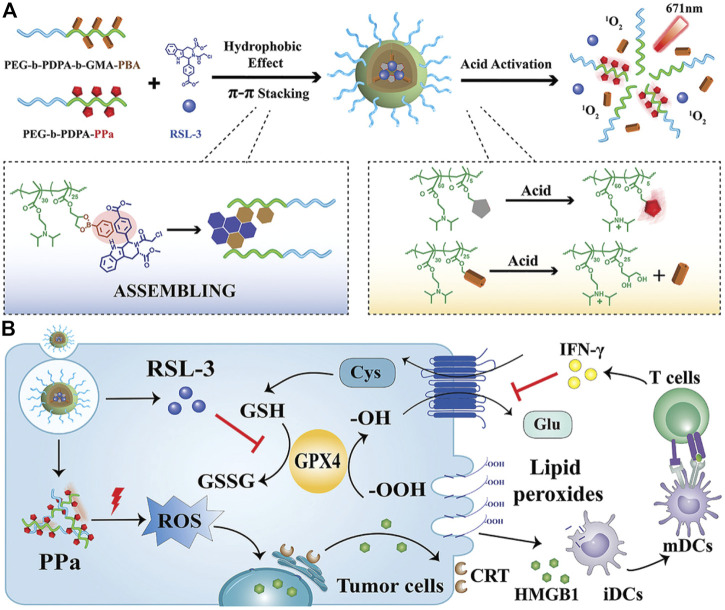
**(A)** Synthesis scheme of NPs. **(B)** Schematic illustration of the mechanism. Adapted from Song et al., (2021) Copyright @ 2021 (Wiley).

RSL3 can be loaded onto the non-iron nanomaterials. The ionizable block copolymer and phenylboronate ester (PBE) were integrated by covalent bonds to prepare a platform for RSL-3 delivery (Figure 14) (Song et al., 2021). At neutral pH, the NPs could stably embed RSL-3 in the hydrophobic core through π–π interaction with the PBE groups and release the payload in the endogenous vesicles through acidity-triggered cleavage of dynamic covalent bonds of PBE. In addition, the NPs could perform acid-activatable PDT by ionizing nuclear protonation and significantly recruiting tumor infiltrating T lymphocytes to secrete IFN-γ, and thus make the B16–F10 melanoma tumor sensitive to RSL-3–induced ferroptosis. The maltose ligand and azobenzene were linked by PEG and self-assembled to load RSL3, yielding Malt-PEG-Abz@RSL3 (Li et al., 2022). Malt-PEG-Abz@RSL3 micelles could actively target HepG2 because of the high expression of glucose transporters, thus achieving RSL3 delivery. Malt-PEG degraded to release RSL3 for inhibiting GPX4 activity, whereas NADPH that took part in synthesis of GSH and thioredoxin-SH2 [Trx (SH) 2] was consumed by the azobenzene moiety, resulting in the reduction of GSH and Trx (SH) 2. The azobenzene was also connected to a nitroimidazole-conjugated polypeptide and self-assembled with RSL3 (Guo, X. et al., 2020). Under anoxic conditions, the azobenzene moiety caused PEG to be removed and reinforced micelle uptake in 4T1 cells, while the nitroimidazole moiety not only resulted in the rapid release of RSL3 but also exhausted intracellular NADPH with the help of overexpression of nitroreductase in the tumor site.

#### 3.2.6 UC Nanomaterials

**FIGURE 15:**
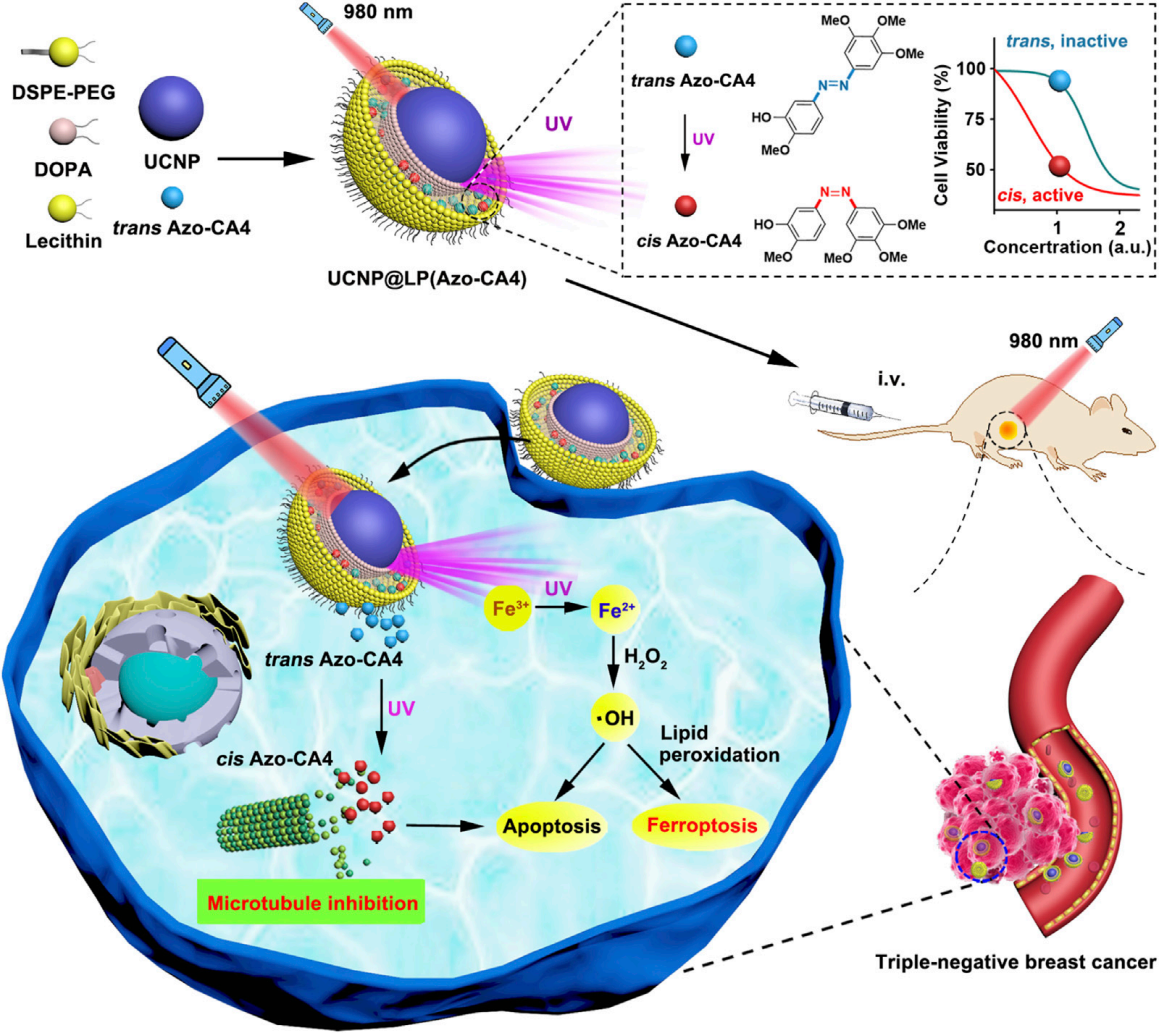
Synthesis scheme and mechanism of UCNP@LP (Azo-CA4). Adapted from Zhu et al., (2020) Copyright @ 2020 (ACS).

The core-shell UCNPs (NaGdF4:20%Yb, 0.5%Tm^3+^@NaGdF4) could absorb NIR light and emit UV to transfer azobenzene combretastatin A4 (azobenzene-CA4) to activate azobenzene-CA4, while UV light could induce reduction of intracellular Fe^3+^ to Fe^2+^ (Figure 15) (Zhu et al., 2020). Upon NIR irradiation, the viability of the triple-negative breast cancer cells treated with nanocarriers loaded with azobenzene-CA4 evidently decreased. Another UCNP (NaYF4:20%Yb, 2%Er@NaYF4) was coated by the MS and liposome to encapsulate Ce6 and buthionine sulfoximine (BSO) (Li D et al., 2021; Li Z et al., 2021). Notably, Ce6-induced ROS production, BSO-caused GSH depletion, and inactivation of GPX4 could amplify apoptosis and ferroptosis. Efficient tumor destruction led to increased exposure of high mobility group box 1 (HMGB1) and calreticulin enhancing antitumor immune responses, including maturation of DCs and effector function of tumor-infiltrating T lymphocytes, which resulted in the elimination of the residual melanoma cells.

## 4 Conclusions and Future Perspectives

With the development of life sciences and medicine, the genetic and molecular mechanisms of cancer have been extensively investigated by researchers worldwide. Many countries have invested considerable material and financial resources in this endeavor. However, tumorigenesis, progression, metastasis, recurrence, and death caused by cancer are still poorly understood because of the tumor complexity including diverse molecular types and heterogeneity; thus, the therapeutic outcomes remain unsatisfactory. Therefore, finding novel therapeutic strategies to solve the human cancer issue has become the most significant problem that needs to be urgently addressed to protect human health. Ferroptosis, termed in 2012, has been widely researched concerning the cellular mechanisms and signaling pathways in cancers. As a novel type of cell death, ferroptosis has great potential as a new cancer therapy, and several ferroptosis-inducing small molecules (such as erastin, sorafenib, sulfasalazine, altretamine, and artemisinin) and various genes related to ferroptosis (e.g., xCT, GPX4, p53, NQO1, and NOX) have been discovered by the researchers. Nonetheless, there are also some disadvantages: 1) there are potential adverse effects of ferroptosis on normal tissues due to the low specificity of small-molecule compounds; 2) small-molecule compounds have short half-lives in blood and low accumulation at tumor sites because of rapid renal clearance; 3) gene-based FBT for cancer is essentially one type of gene therapy, which might bring controversy at ethics and law (Shen et al., 2018). Hence, although FBT based on small molecules and genes is promising, significant research is required before it can be applied clinically.

With the rapid development of nanomedicine, numerous ferroptosis-inducing nanomaterials have been developed by chemists, pharmacists, and material scientists. The adverse effects on normal tissues could be decreased because of the precise targetability of the nanomaterials to tumor sites, and the nanomaterials possess long circulating half-lives in the blood, which enhances the accumulation of the nanomaterials at tumor sites. The ferroptosis-based nanomaterials could be triggered by several factors (such as light, US, and TME) to induce the release of metal irons, metal oxides, or drugs to induce or enhance ferroptosis. The FBT of cancer could integrate with different models of imaging guidance (such as US imaging, PAI, and MRI) and combine with diverse cancer therapies (such as CDT, SDT, PDT, PTT, and immunotherapy). In this review, we roughly divide the reported ferroptosis-based nanomaterials into two types: iron-contained nanomaterials and iron-free nanomaterials. The former induces ferroptosis *via* increasing the external iron to enhance the Fenton reaction; meanwhile, the latter induces ferroptosis *via* GSH depletion, GPX4 inactivation, or increasing intracellular ROS, which leads to the prospective that it is difficult for the iron-free nanomaterials to achieve satisfactory treatment. Therefore, iron-contained nanomaterials might be more promising than iron-free nanomaterials because the latter is limited by utilizing the intracellular restricted iron to produce ROS *via* the Fenton reaction.

Undoubtedly, in the future, a mass of ferroptosis-based nanomaterials will be investigated for cancer therapy. The future research might focus on the following aspects: 1) more ferroptosis small-molecular compounds will be discovered and synthesized, and subsequently they might be utilized to be encapsulated into the iron-contained nanomaterials for FBT; 2) the siRNAs targeting ferroptosis-related genes (e.g., p53, GPX4, and xCT) might be used to enhance the effect of FBT; 3) the ferroptosis-based nanomaterials might combine with radiotherapy to make tumor sensitized to radiation; 4) more nanomedical research about the combination treatment of FBT and immunotherapy needs to be brought to the forefront because of its great clinical application prospects. Meanwhile, multiple issues should be taken into consideration in the rational design of the nanomaterials based on ferroptosis in the future, such as safety and feasibility of clinical application, possible toxicity, and industrial feasibility.
